# Simplified Fréchet Distance for Generative Adversarial Nets

**DOI:** 10.3390/s20061548

**Published:** 2020-03-11

**Authors:** Chung-Il Kim, Meejoung Kim, Seungwon Jung, Eenjun Hwang

**Affiliations:** 1 School of Electrical Engineering, Korea University, Seoul 02841, Korea; cilkim1@korea.ac.kr (C.-I.K.); jsw161@korea.ac.kr (S.J.); 2 Research Institute for Information and Communication Technology, Korea University, Seoul 02841, Korea; meejkim@korea.ac.kr

**Keywords:** image processing, generative models, generative adversarial net

## Abstract

We introduce a distance metric between two distributions and propose a Generative Adversarial Network (GAN) model: the Simplified Fréchet distance (SFD) and the Simplified Fréchet GAN (SFGAN). Although the data generated through GANs are similar to real data, GAN often undergoes unstable training due to its adversarial structure. A possible solution to this problem is considering Fréchet distance (FD). However, FD is unfeasible to realize due to its covariance term. SFD overcomes the complexity so that it enables us to realize in networks. The structure of SFGAN is based on the Boundary Equilibrium GAN (BEGAN) while using SFD in loss functions. Experiments are conducted with several datasets, including CelebA and CIFAR-10. The losses and generated samples of SFGAN and BEGAN are compared with several distance metrics. The evidence of mode collapse and/or mode drop does not occur until 3000k steps for SFGAN, while it occurs between 457k and 968k steps for BEGAN. Experimental results show that SFD makes GANs more stable than other distance metrics used in GANs, and SFD compensates for the weakness of models based on BEGAN-based network structure. Based on the experimental results, we can conclude that SFD is more suitable for GAN than other metrics.

## 1. Introduction

Generative Adversarial Net (GAN) is one of the models drawing attention in the field of machine learning (ML) and computer vision [1]. The model learns the distribution of a given data and generates sample data based on the learning.

Recently, several GAN models have been proposed to deal with different purposes, and the performances of generative models have improved. For instance, Domain Adversarial Neural Network (DANN) and Adversarial Discriminative Domain Adaption (ADDA) considered image-to-image translation [2,3] and GAN with text manifold interpolation and image-text matching discriminator considered text-to-image synthesis [4]. Super-Resolution Generative Adversarial Nets (SRGAN) focused on super-resolution [5], and style transformation [6,7], Context Encoder (CE) and Globally and Locally Consistency Image Completion (GLCIC) considered inpainting [8,9], and Generative Adversarial Nets for Video generation (VGAN) was applied to generate high-dimensional data based on image, video, and audio [10].

The principle of GANs is to set up a game between two players: a generator and a discriminator. The generator generates samples based on the distribution obtained from training data [11]. On the other hand, the discriminator examines whether the input sample is real or fake when a data sample is given. During the training of a model, the generator is trained to deceive the discriminator, while the discriminator is trained to distinguish the generated samples from the real samples correctly. The optimal generator generates plausible data samples, and this makes the discriminator foolish and unable to work eventually [12]. Owing to many efforts to improve GANs [13,14,15], the data generated by GANs are so realistic that human beings almost cannot differentiate real data from fake data.

Each loss of generator and discriminator should converge to a constant during the training process for success data generation. If this occurs, then the training process of the GAN is called “stable” [13]. Usually, GANs suffer from unstable training for several reasons. Examples of unstable phenomena include the vanishing the gradient, the gradient becoming too large, or the loss oscillating during training [12,13]. Another problem that GAN is experiencing is the generator collapsing, which produces only a single sample or a small family of very similar samples. These phenomena are called mode collapse and mode drop. Mode collapse is generating the similar or even the same outputs for different random input vectors, while mode drop is concerned with modes being dropped from the output distribution [1]. These phenomena may occur when the distribution of real data cannot be represented correctly because of using inadequate optimizations or insufficient network resources that cause an inability of node counting [1,13,14]. In such conditions, the average of real data distribution is used for mode collapse, while the distribution of real data is ignored for mode drop, during the generation of fake data.

To be an acceptable GAN, the distance between the two distributions of real data and generated data has to be far in the discriminator’s viewpoint, while it has to be near the generator’s viewpoint. The performance of a GAN, therefore, is closely related to the adopted distance metric in the loss functions, and the instability of networks might be solved by changing the distance metric. One of the studies of this approach was the Wasserstein Generative Adversarial Network (WGAN) [15]. Unlike the original GAN, WGAN applied Wasserstein-1 distance, called Earth Mover’s distance (EMD), to measure the distance between two distributions of real data and generated data [16]. The superiority of EMD was considered in terms of stability and quality of a GAN by comparing these values of EMD with those of other distance metrics involved. For instance, WGAN compared EMD with Jensen–Shannon distance (JSD) and Kullback–Leibler (KL) divergence [17,18]. With the advent of WGAN, EMD has been used widely in GANs as a metric in loss functions.

Recently, Fréchet distance (FD), called as Wasserstein-2 distance, has been introduced [19]. FD was initially used as a similarity evaluation index, called Fréchet Inception distance (FID), like the Inception Score (IS) [20]. FID that applied FD was used in the inception v3 model [21]. It used feature values extracted from a pre-trained inception v3 model to evaluate the similarity of real data and generated data [22]. On the other hand, IS was a score correlating human judgment with a pre-trained ImageNet dataset in inception v3 networks [23]. An experiment was conducted to verify the adequateness of FID as an evaluation index, and the superiority of FID over IS demonstrated [20]. Therefore, FID became a primary evaluation index used in GANs [24,25,26]. However, it is very challenging to apply FD in GANs directly because it requires a longer time and bigger memory compared to other distance metrics [27,28], which is caused by its complexity. To the best of our knowledge, there is no GAN using FD as a metric of training GAN until so far.

In this paper, we introduce a Simplified Fréchet distance (SFD) and propose a GAN model in which SFD is involved. SFD is a simplified and regularized version of FD. That is, SFD reduces the complexity of FD and enables the training process stable representing some of the characteristics of FD. Therefore, a portion of the characteristics of FD could be explored when SFD is used in the training process of GANs. The structure of the proposed GAN model, the Simplified Fréchet Generative Adversarial Networks (SFGAN), is based on the Boundary Equilibrium GAN (BEGAN) [29]. The difference between the two GANs is the distance metric used in the loss functions of the networks. SFGAN uses SFD, while BEGAN uses EMD. In other words, SFGAN is trained by adversarial losses that are defined by SFD among the distributions of input and output. Output distribution is computed through auto-encoder based discriminator by using an adversarial loss.

For demonstrating the superiority and applicability of SFGAN, the experiments are conducted with the CelebA, CIFAR-10, and a 2-D mixture of Gaussians [23,30,31]. Two purposes are considered in the experiment. One is to investigate the stability of training GANs by using SFD and EMD, and the other is to compare JSD, EMD, and SFD between real data and generated data during the training procedure of SFGAN and BEGAN. The trainings are executed up to three million steps to investigate the stability of training and the differences in distance metrics before and after mode collapse and/or mode drop. The same values of hyperparameters are used in the two models. The experiment is conducted five to ten times to see whether the results change with each experiment. It is observed that the differences in results for all experiments are negligible. Experimental results show that the training process of SFGAN seems stable, and neither mode collapse nor mode drop is detected. On the other hand, these phenomena have occurred during the training process of BEGAN, which result in unstable training. Moreover, it is observed that SFD distinguishes the distributions of real data and fake data generated by unstable BEGAN, while EMD sometimes fails it.

The contributions of this study are summarized as follows: (1) A Simplified Fréchet distance is introduced. SFD reduces the complexity of FD, representing some characteristics of FD and enables stable training by compensating the weakness that the models belonging to BEGAN-based network structure have. (2) A new GAN model in which SFD is involved in the loss functions, SFGAN, is proposed. SFGAN is more stable than BEGAN in which EMD applied. (3) SFD is introduced as an evaluation index for detecting mode collapse and/or mode drop during the procedure of training a GAN. It is possible to detect instability of a GAN during the training with SFD alone, without requiring additional models, a balanced dataset or constrained space that other GANs are requiring.

This paper includes the following: The related works are presented in Section 2. In Section 3, we introduce SFD and compare it with the existing distance metrics. The SFGAN model is presented in Section 4, and the stability and effectiveness of the SFGAN model are verified via experiments in Section 5. Lastly, Section 6 concludes the paper.

## 2. Related Works

**Figure 1:**
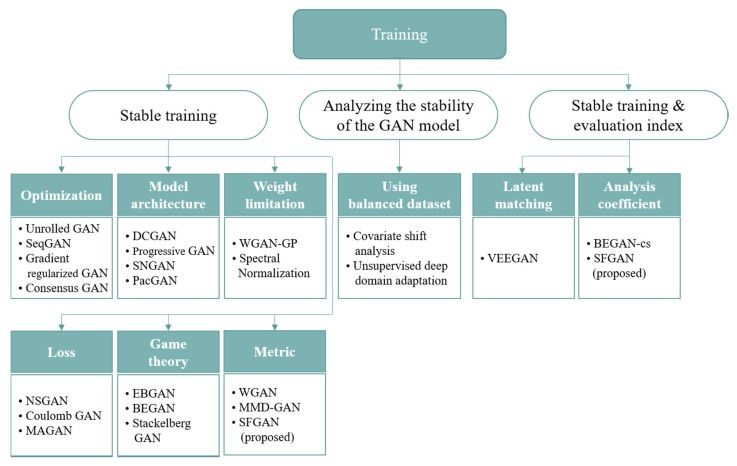
Categorization of studies on Generative Adversarial Networks (GANs) that deal with stability.

There are many studies on GANs [13,14,15,24,32,33,34,35,36,37,38,39,40,41,42,43,44,45,46,47]. In this section, we investigate the studies that consider the stability problem during the training of GANs. The studies dealing with the stability on GANs can be divided into three categories: the GANs that consider stable training by evaluating performances with only IS or FID, the methodologies to analyzing the stability of the GAN model, and the GANs that consider stable training with their own evaluating methods to investigate the stability. Figure 1 presents the known studies for the three categories.

### 2.1. Stable Training

Many studies are dealing with stable training of GANs [13,14,15,24,32,33,34,35,36,37,38,39,40,41,42,43]. Notably, several studies [13,14,15,32,33] also considered on mode collapse and/or mode drop problems. In these studies, IS or FID is used as a performance evaluation index. This category can be classified further according to their purposes or applied methods: optimization, model architecture, weight limitation, loss, and game theory.

#### 2.1.1. Optimization

The studies in the optimization class deal with the optimization of balancing of the generator and discriminator for stable training. Unrolled GAN, SeqGAN, Gradient regularized GAN, and Consensus GAN belong to this class [13,14,32,33].

The network parameters of the generator in the unrolled GAN are updated according to gradient descent (GD) of every step, which reflects the state from the current step to some fixed number of future steps, while those parameters of discriminator are updated according to GD of current step only. Based on the updating rule, the unrolled GAN can mitigate the mode collapse and stabilize the training of GANs. On the other hand, SeqGAN modeled a generator as a stochastic policy in reinforcement learning (RL). SeqGAN performs the gradient policy update directly to avoid the difficulty of differentiation of discrete data such as text generation and music generation. It showed that the performance of SeqGAN outperforms that of the original GAN. A scheme imposing a regularization penalty during the generator update was proposed [32]. This scheme was adopted in the original GAN, and the local stability was confirmed. This local stability is updating the gradient near an equilibrium point. Two major failures caused by GD optimization, vanished real-part and large imaginary-part in the eigenvalues of the Jacobian, were considered in Consensus GAN [33]. By using their Consensus Optimization, Nash equilibrium was found by more powerful optimization than GD based optimization. Nash equilibrium is a state that no player in this state can gain more rewards by changing its own strategy [48]. If the generator and the discriminator reach a Nash equilibrium state, the objective of GANs does not change any more theoretically, so this theory keeps the stability of GANs during the training.

#### 2.1.2. Model Architecture

The GANs in the model architecture class considered network architectures and their parameters for stable training. Deep convolutional GAN (DCGAN), progressive GAN, spectral normalization GAN (SNGAN), and packed GAN (PacGAN) are belonging to this class [24,34,35,36].

DCGAN is a Convolutional Neural Network (CNN)-based GAN model considering the way of setting parameters and techniques for optimizing GAN [34]. It dealt with the use of batch normalization and activation function. The resolution of images was increased up to 1024 by adding layers, and mini-batch was also considered to improve stability in progressive GAN [24]. SNGAN considered an architecture utilizing the residual block [35,49], and PacGAN considered to use an augmented discriminator. The discriminator in PacGAN maps multiple samples that are jointly coming from either real data or the generator to a single label [36].

#### 2.1.3. Weight Limitation

In the weight limitation class, several techniques are dealing with the discriminator’s weights in a network’s nodes. The techniques are schemes to control the instability of GANs.

Gradient-penalty and spectral normalization are typical examples in this class [37]. The main idea of WGAN gradient-penalty (WGAN-GP) is considering the Lipschitz-1 constraint in WGAN. The gradient penalty was added to the loss of WGAN, which is directly constraining the gradient norm of the discriminator. By adjusting this method, WGAN-GP outperformed original WGAN in terms of stable training and similarity between real data and generated data. SNGAN belonging to the model architecture class also proposed a spectral normalization technique to increase the stability of training [34]. Unlike gradient-penalty, spectral normalization does not depend much on the current generative distribution but regularizes the weights of nodes in a network. Training with spectral normalization was compared with that of gradient-penalty, and it was concluded that the former does not easily destabilize with a high learning rate while the latter destabilizes.

#### 2.1.4. Loss

Other recent approaches are considering new loss functions. NSGAN considered a risk that the gradient of the generator would vanish when the original GAN loss is used [12]. For preventing the risk, a function was proposed to maximize the loss of generator, and better performance was obtained than that of the original GAN models. Margin adaption for GAN (MAGAN) evaluated the performance improvement of stable training by using an adaptive hinge loss, which estimates the appropriate margin of the loss [38]. MAGAN not only generated diverse datasets but also achieved an improvement in terms of IS compared to energy-based GAN (EBGAN) and boundary equilibrium GAN (BEGAN) [29,39]. Coulomb GAN trained the networks using the Coulomb potential equation that makes samples attracted to the training samples but repulsed to each generated sample [40]. It was shown that Coulomb GAN has only one Nash equilibrium.

#### 2.1.5. Game Theory

In the game theory class, most studies used the Nash equilibrium [48]. For instance, EBGAN and BEGAN considered Nash equilibrium [29,38]. These two models generate realistic data successfully and hardly fail to learn the distribution of data. Stackelberg GAN was inspired by the Stackelberg competition of game theory [41]. It is known that the Stackelberg model can be used to find the perfect Nash equilibrium of sub-games. Experiments verified the effectiveness of the Stackelberg competition by using a multi-generator architecture.

#### 2.1.6. Metric

The other approach of dealing with instability is considering distance metrics used in GANs. As far as we know, Wasserstein GAN (WGAN) is the first study to improve the learning stability of GANs by defining a new distance metric between data distributions [15]. It was shown that traditional distances such as JSD are insufficient for data training in GANs. As an approach to mitigate this problem, EMD was applied to WGAN. MMD-GAN used a maximum mean discrepancy (MMD) as a distance and adopted auto-encoder [42,43]. The definition of MMD can be found in Appendix A. This model’s discriminator was trained via MMD with adversarially learned kernels. Although it was obtained that the IS of MMD-GAN was higher than WGAN when the experiment was conducted with CIFAR-10, there are two problems in this model: (i) The performance of MMD-GAN comes only by using either per-pixel reconstruction error term or gradient penalty. (ii) The fine learning from data seems to discourage to contract the discriminator outputs of real data using MMD [50].

Improving distance metrics in GANs has the following advantages: (1) No additional network models may be required for stable training a GAN, and existing losses can be used as they are. (2) The state of mode collapse and/or mode drop during the training process can be identified through the proposed metric. The training curves and sample graphs by WGAN and MMD-GAN showed the relation between the loss and the sample quality. As our study proposes a metric, the proposed model can also take the advantages that these studies grouped by this section have.

### 2.2. Analyzing the Stability of the GAN Model

Although analyzing the stability of the GAN model did not affect directly stable training of GANs, studies in this category detected and showed these phenomena based on the data generated by the trained model. Covariate shift analysis was a scheme to add a multi-class classifier in a balanced multi-class dataset to investigate whether the data generated by GAN was biased [44]. Unsupervised deep domain adaptation was a scheme to extend covariate shift analysis to an unbalanced dataset with the existence of a balanced dataset [45].

### 2.3. Stable Training and An Evaluation Index

In this category, there are several studies recently. For instance, an algorithm was proposed in Variational Encoder Enhancement GANs (VEEGAN) to estimate mode collapse [46]. It was conducted by training a multi-layer neural network with sample data and the standard deviation of the data. Boundary Equilibrium Generative Adversarial Nets-Constrained Space (BEGAN-CS) added an embedding space-constrained loss in BEGAN and showed the stability improvement by using the proportional coefficient’s variation during training [47]. Although the results of VEEGAN and BEGAN-CS were noticeable, they had limitations that require additional CNN models and a constraint of latent space for a discriminator, respectively.

Our model, SFGAN, detects mode drop and (or) mode collapse during training. The details of SFGAN are presented in Section 3. Experimental results of SFGAN and BEGAN presented in Section 5 show that the output values of the two models are related to the mode drop and (or) mode collapse of BEGAN.

## 3. Simplified Fréchet Distance

In this section, we introduce SFD and present its advantages. Section 3.1 defines notations to describe image distribution and then introduces the Fréchet distance and SFD based on the defined notations. The advantages of SFD are investigated in Section 3.2 by comparing it with other distance metrics using two different examples.

### 3.1. Simplified Fréchet Distance

We introduce distance metrics of image distributions. For defining distance metrics, images have to be converted to numerical values. Consider a color image that has h and w pixels for height and width, respectively. As an image usually consisted of three channels, R, G, and B, and its values are numbers, without loss of generality, we assume that each pixel of an image has a number for each channel. Then, the image has 3hw pixels in total. Let X be a random vector whose components consist of a random variable $X_{i,j}^{c},$ where $X_{i,j}^{c}$ is the value of pixel (i,j) for c= R, G, B. Then X can be written as

(1) $$X=(X_{i,j}^{c}),i=1,\cdots ,h,\;j=1,\cdots ,w,\;c=R,\;G,\;B.$$

**Figure 2:**
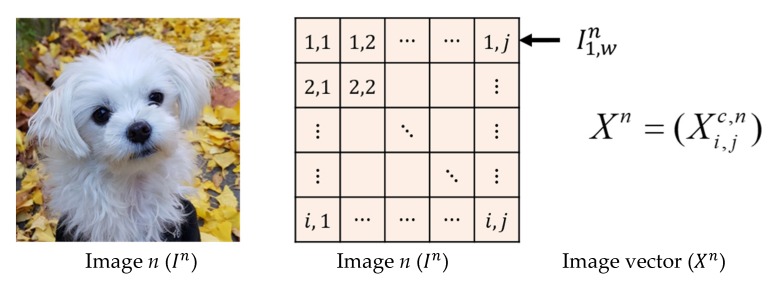
Conversion of an image to an image vector.

From now on, we call X as an ‘image vector’ and describe distance metrics in terms of the image vectors. Figure 2 illustrates the way of converting an image to an image vector.

We first describe FD of images and then introduce SFD. Assume that there are k images and denote an image vector corresponding to the *n*-th image as $X^{n}$. Then, $X^{n}$ is given by $X^{n}=(X_{i,j}^{c,n}),\;i=1,\cdots ,h,\;j=1,\cdots ,w,\;c=R,\;G,\;B.$

Let X and Y be random vectors given by image vectors and F and G be their distributions, respectively. Let $m_{•}$ and $C_{•}$ be the mean vector and covariance matrix of a variable •, respectively, where •=X,Y. Then, $m_{X}$ and $C_{X}$ of X are defined by

(2) $$\begin{array}{c} m_{X}=(m_{X_{i,j}^{c}})=\left(\frac{1}{k}\sum_{n=1}^{k}X_{i,j}^{c,n}\right)\;\mathrm{and}\;C_{X}={\left(X_{i,j}^{c}-m_{X_{i,j}^{c}}\right)}^{T}(X_{i,j}^{c}-m_{X_{i,j}^{c}}),\\ i=1,\cdots ,h,\;j=1,\cdots ,w,\;c=R,\;G,\;B. \end{array}$$

respectively, where T in $C_{X}$ represents transpose of a matrix.

**Definition** **1.** *Fréchet Distance*.

The Fréchet distance Fr(F,G) between two distributions F and G is defined by

(3) $$Fr^{2}\left(F,G\right)=\underset{X,Y}{\min}E{\left|X-Y\right|}^{2},$$

where E represents the expectation, and the minimization is taken over all random variable X and Y having distributions *F* and *G*, respectively [19].

In particular, if X and Y follow multivariate normal distributions $Fr^{2}\left(F,G\right)$ is given by

(4) $$Fr^{2}\left(F,G\right)={\left\|m_{X}-m_{Y}\right\|}^{2}+\mathrm{tr}\left\{C_{X}+C_{Y}-2{\left(C_{X}C_{Y}\right)}^{1/2}\right\},$$

where ‘tr’ in Equation (4) represents the trace of a matrix [51].

FID was a metric providing a better result for measuring the similarity between the two distributions of generated data and the real data. For instance, FID was compared with the IS in experiments using various data [20]. However, the covariance term in Equation (4) has drawbacks in adopting FD as a loss to train GAN. These drawbacks were demonstrated in [28] by the empirical results using the MNIST [52], Fashion-MNIST [53], CIFAR-10 [31], and CelebA [30] datasets. In [28], each dataset was divided into two groups, and the FID was used as the similarity index between the groups. It was observed that estimating a total covariance matrix can be unnecessary and unproductive. It was also mentioned that a constrained version of FID might be enough to represent distances between data. Based on this, it seems relevant to delete the covariance term in FID. By considering this aspect, it may not be a problem to apply the distance metric without covariance in FD to the data, not the inceptionv3 feature of data. Furthermore, the larger the dimension size of the datasets is, the higher the computational load on the covariance matrix is. These facts motivate SFD. That is, SFD simplifies and regularizes the covariance term in FD to reduce the complexity of FD and to learn stably, respectively. This distance metric makes applicable FD in the training process of GANs with less computing load. For this purpose, we assume that all components of both random vectors X and Y are independent. There is no guarantee that these components are independent, and they may be dependent on the real world. In the field of deep learning, however, such an assumption was used in several studies, and the better results were obtained under the assumption [54,55,56]. The independence of the two variables does not imply that they have the same variances. The SFD is introduced with this assumption.

**Definition** **2.** *Simplified Fréchet Distance*.

The Simplified Fréchet distance SF(F,G,α) between two multivariate normal distributions F and G with coefficient α is given by

(5) $$SF^{2}\left(F,G,\alpha \right)={\left\|m_{X}-m_{Y}\right\|}^{2}+\frac{1}{\alpha }{\left\|\sigma_{X}^{}-\sigma_{Y}^{}\right\|}^{2},$$

where $\sigma_{•}^{2}$ is the variance of a random variable • and α is a constant for regularization.

In the following, SF(F,G,α) is representing $\sqrt{SF^{2}\left(F,G,\alpha \right)}$.

### 3.2. Advantages of Simplified Fréchet Distance

To investigate the advantages of SFD, we consider two examples. Although these examples might be extreme cases, they can appear in the training process of GANs. For the two examples, SFD is compared with two distance metrics: JSD and EMD. The definitions of JSD and EMD can be found in Appendix A.

**Example** **1.** *(Learning parallel distribution) The distributions of real data and estimated data are parallel.*

It was shown that JSD is inadequate, while EMD is adequate to measure data distributions that are parallel [15]. We consider the two-dimensional random vectors X and Y whose components are normally distributed with means (0,0) and (θ,0), and covariance matrices $\left(\begin{array}{cc} 0 & 0\\ 0 & 1 \end{array}\right)$ and $\left(\begin{array}{cc} 0 & 0\\ 0 & 1 \end{array}\right)$, respectively. Then, the distributions of X and Y are parallel.

**Example** **2.** *The distributions of real data and estimated data are a couple of univariate normal distributions with mean zero.*

Let X and Y be the one-dimensional random variables distributed according to normal probability density function (pdf) with means 0 and 0, variances 1 and $\delta^{2}$, respectively.

**Figure 3:**
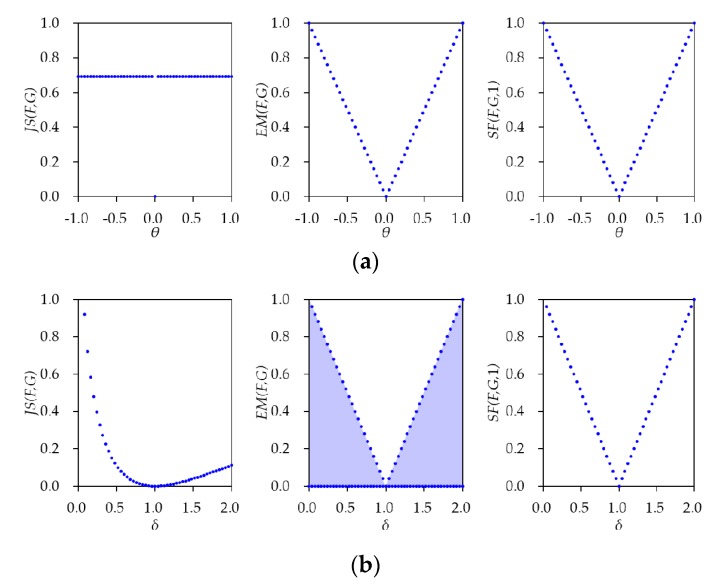
Description of Jensen–Shannon distance (JSD), Earth Mover’s distance (EMD), and Simplified Fréchet distance (SFD) with α=1 for the examples: (**a**) Example 1 for θ∈[−1,1]; (**b**) Example 2 for δ∈[0.04,2].

The three distances for the two examples are represented in Table 1. The detailed derivation of the obtained values can be found in Appendix B. According to the table, JSD is a constant or log2, regardless of θ,θ≠0. On the other hand, EMD and SFD are varying according to θ for Example 1. These values imply that JSD cannot distinguish the given distributions, while EMD and SFD can distinguish those. For Example 2, on the other hand, EMD has zero as a lower bound regardless of δ, while JSD and SFD depend on the δ. That is, EMD cannot distinguish the given distributions, while JSD and SFD can distinguish those. Figure 3 illustrates the obtained three distances given in Table 1 for varying θ and δ with α=1 for SFD; (a) Example 1 with θ∈[−1,1], (b) Example 2 with δ∈[0.04,2]. From the two examples, it is noticed that SFD is the only distance metric that can always be expressed in terms of the respective parameters θ and δ.

This disadvantage of EMD is not limited to a univariate normal distribution, as shown in Example 2. By Equation (A22), it is noteworthy that the lower bound of EMD is zero for other cases, such as the given two distributions are multivariate normal distributions with the same mean but other than zero. In this case, EMD cannot distinguish the two distributions.

## 4. Simplified Fréchet GAN

In this section, we present a GAN model, simplified Fréchet GAN (SFGAN). SFGAN uses the SFD in the calculation of the discriminator loss and the generator loss in a GAN.

Since GANs are trained by an adversarial loss that is based on the discriminator output, the output must have a multivariate normal distribution to apply SFD. As far as we know, however, no studies have considered multivariate normal distributions as outputs. It is widely accepted in the image processing field that the distribution of a lot of images is assumed as multivariate normal [57,58,59], and the distribution of output data will be the same as that of input data through an auto-encoder. For this reason, we design the discriminator in the form of an auto-encoder.

Three candidates can be considered for the baseline of our model; BEGAN, EBGAN, and MMD-GAN. The architectures of all three models include an auto-encoder. However, EBGAN cannot apply measures based on data distributions because EBGAN uses errors per pixel. Therefore, EBGAN is excluded from the candidates. As MMD-GAN requires additional reconstruction error term, the error has to be defined additionally as the form of per-pixel error. Therefore, this model is not relevant to the baseline model. Since BEGAN is only required to replace distance metric, it seems to be the better model than the other two models.

**Figure 4:**
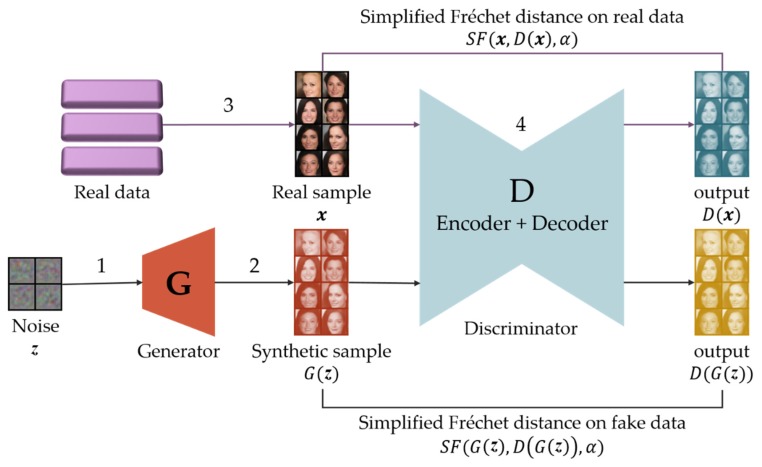
Schematic diagram of the SFGAN algorithm. The numbers on the arrow indicate those steps in the procedure of SFGAN.

For this reason, we select BEGAN as the baseline model of SFGAN. The network architecture and used losses in the network of SFGAN are the same as those of BEGAN. The only difference is the used distance metric, SFD for SFGAN, and EMD for BEGAN. Figure 4 illustrates the model architecture and procedure of SFGAN. The procedure, including the data flow of SFGAN, is described in Table 2.

**Figure 5:**
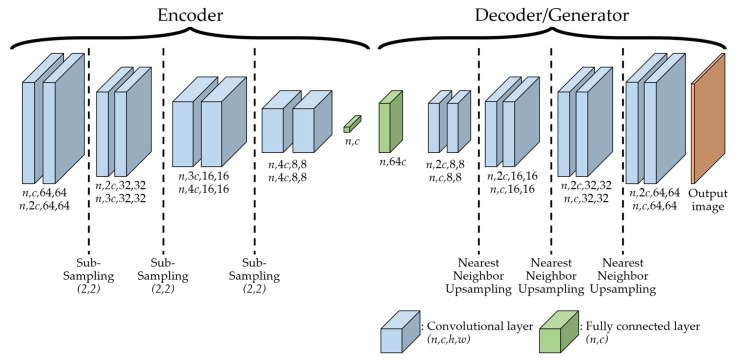
The architecture of SFGAN for discriminator and generator in 64 × 64 resolution with batch size *n* and filter number *c*.

The detailed structure in D will be explained according to Figure 5. The loss functions in SFGAN are as same as those of BEGAN except involved distance metric in the functions. The losses consist of discriminator loss $L_{D}$ and generator loss $L_{G}$, which is given by

(6) $$L_{D}=E_{x\sim p_{data}(x)}\left[SF\left(x,D(x),\alpha \right)\right]-k_{t}E_{z\sim p_{Z}(z)}\left[SF\left(G(z),D\left(G(z)\right),\alpha \right)\right]$$

and

(7) $$L_{G}=E_{z\sim p_{Z}(z)}\left[SF\left(G(z),D\left(G(z)\right),\alpha \right)\right],$$

where $E_{r\sim p}$ represents the expectation of variable r with pdf p. $k_{t}$ is a variable for stable learning at *t* step which is updated by proportional control given by

(8) $$k_{t+1}=k_{t}+\lambda_{k}\left(\gamma \left(SF\left(x,D(x),\alpha \right)\right)-SF\left(G(z),D\left(G(z)\right),\alpha \right)\right),$$

where $k_{0}=0$, $k_{t}\in [0,1]$, $\lambda_{k}$ is the proportional gain depending on $k_{t}$, and γ is a hyperparameter for controlling image diversity taking values in the interval [0,1]. Note that the small value of γ gives low diversity of generated data. Both of discriminator and generator are trained using the GD method with values of Equations (6) and (7), respectively.

Figure 5 illustrates the overall network architecture of SFGAN. This architecture consists of two parts: encoder and decoder/generator. The decoder and the generator have the same structure with different weights. The discriminator consists of an encoder and a decoder. All convolutional layers in the encoder are the same as the layers in the generator except for the number of filters. In the sub-sampling layers of the encoder, the input of a layer is downsampled using stride two, which reduces the input size as it passes through the layers. On the other hand, in the up-sampling layers of the decoder/generator, the input of a layer is up-sampled using the nearest neighbor method, which enlarges the input size as it passes through the layers.

The input of encoder is the real data or the generated data, while the input of decoder is the output of the encoder, which is called as a hidden variable. The input of the generator is the noise sample vectors z and it gets into the fully connected layer. All convolutional layers use 3×3 convolutions with exponential linear units [60] and are repeated twice for better output. The batch size of the discriminator is twice of that for generator because discriminator has to afford to deal with both real data and generated data. In the figure, n and c represent the batch size and the number of filters, respectively.

## 5. Experiments

In this section, we present experimental results. We implemented two experiments. One is for stability comparison of generation models, and the other is for comparison of distance metrics to detect mode collapse and/or mode drop during training the network. For performance comparison, BEGAN is executed in the experiment. SFGAN and BEGAN are trained until mode collapse and/or mode drop occur to compare the stability. In the former experiment, the images generated during the training at every 1000 steps, the measured values of losses, and the $k_{t}$s at that time are compared. In the latter experiments, JSD, EMD, and SFD between the generated data and the training data of the two models are measured. As a criterion for the similarity between real data and fake data, FID is considered for the two models.

In the following, the experimental setting is explained in Section 5.1, and the image stability on sequential steps by SFGAN and BEGAN are presented in Section 5.2. The results for verifying the stability of SFGAN and the performance comparison of the two models are presented in Section 5.3 and Section 5.4, respectively.

### 5.1. Experimental Setting

For training, two computers are used. One of them is composed of Intel^®^ CORE™ CPU, NVIDIA GTX 1080ti as GPU with 24 GB RAM, while the other is composed of Intel^®^ Xeon^®^ CPU E5-2680 v4, NVIDIA RTX TITAN as GPU with 128 GB RAM. All experiments are implemented by the TensorFlow library [61].

The CelebA [30], CIFAR-10 [23], and a mixture of Gaussian distributions are used to train the GAN models. These datasets are commonly used in GANs research. The CelebA is a collection of human face images. It is effective at testing qualitative results because human faces are good at recognizing defects [29]. In the experiment, the images of 64 × 64 and 128 × 128 resolutions will be generated for CelebA. The CIFAR-10 is a set of widely used images in the image-based machine learning studies. CelebA has only human faces, and the number of training images is 202,599, while CIFAR-10 contains various images such as trucks, frogs, birds, and ships, which are hardly the same objects, and the number of images is 60,000, relatively small. The last dataset consists of 2-D random variables from the mixture of Gaussian distributions. Eight random variables are distributed in a circle, and each random variable consists of *x* and *y* coordinates. The expectation of each random variable depends on the position of the random variable, and its standard deviation is fixed as 0.02. The test with this dataset was proposed in unrolled GAN [13] to evaluate the performance of discriminator. In the test, it was assumed as unstable if any one of eight distributions are not learned. With the same stability criterion, the stability of SFGAN is also investigated with this dataset.

**Figure 6:**
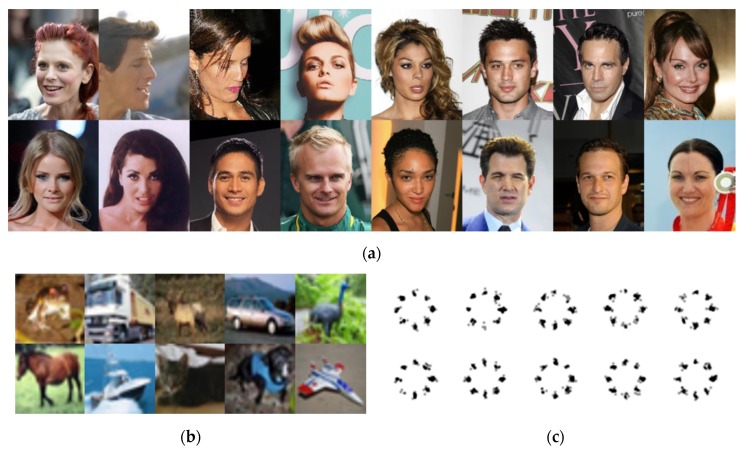
Samples of datasets: (**a**) CelebA; (**b**) CIFAR-10; (**c**) a mixture of Gaussian.

Figure 6 shows samples from CelebA, CIFAR-10, and the mixture of Gaussian. Table 3 summarizes parameters and the corresponding values used in the experiments. Note that the parameter values are the same used in BEGAN-cs [50] or BEGAN. It is noteworthy that two regularization constants are used in the experiments. The value one is for detecting mode collapse and/or mode drop while 12,288 is for training the networks, which is obtained for acceptable learning of 64 × 64 resolution images during the experiments. The experiments are conducted five to ten times to see if the results fluctuate per each experiment. It is observed that the differences among results are negligible. The presented SFD values are one-dimensional values, which are obtained from the experimental results divided by the number of dimensions. Adam is used as an optimizer to both models since this optimizer is invariant to a diagonal rescaling of the gradients [62].

### 5.2. Stability of Training

#### 5.2.1. The Generated Images of CelebA: 64 × 64 Resolution

**Figure 7:**
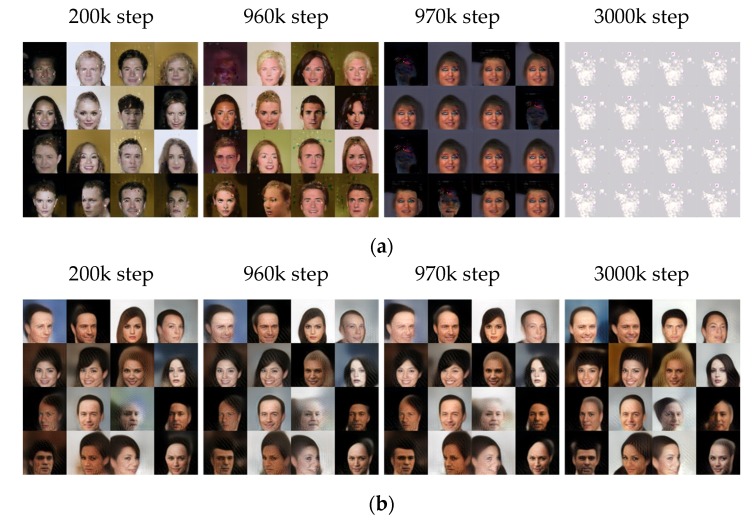
A total of 16 samples generated at 200k, 960k, 970k, and 3000k steps trained with CelebA dataset in 64 × 64 resolution by: (**a**) BEGAN; (**b**) SFGAN with α=12,288.

Figure 7a,b show 16 sample images in 64 × 64 resolution generated in four steps by BEGAN and SFGAN, respectively. 16 samples (i.e., *l* is set to 16) are chosen from a random uniform distribution for the generator’s input at the beginning, and the images generated from the samples are monitored in every 1000 steps until 3000k steps. It is challenging to say mode collapse or mode drop numerically, but the generated samples allow us to determine if the training was as intended. In the 200k step, the training result of BEGAN is similar to that of SFGAN. However, at 970k step, the generated images by BEGAN became weird while SFGAN still generated acceptable human faces. At 3000k step, BEGAN generated very similar images even for different inputs. On the other hand, SFGAN generated diverse human-like face images steadily until 3000k steps. In other words, BEGAN started to generate weird similar face images after 968k steps on the average in a total of ten experiments. This implies that the mode collapse occurs at 968k step, and BEGAN never optimized or restored to stable status after all. On the other hand, SFGAN keeps on generating face images until 3000k steps without mode collapse. Therefore, it can be said that the training process of SFGAN is stable until 3000k steps. However, the example in Figure 7 appears hard to assume that a mode drop has occurred in both BEGAN and SFGAN.

**Figure 8:**
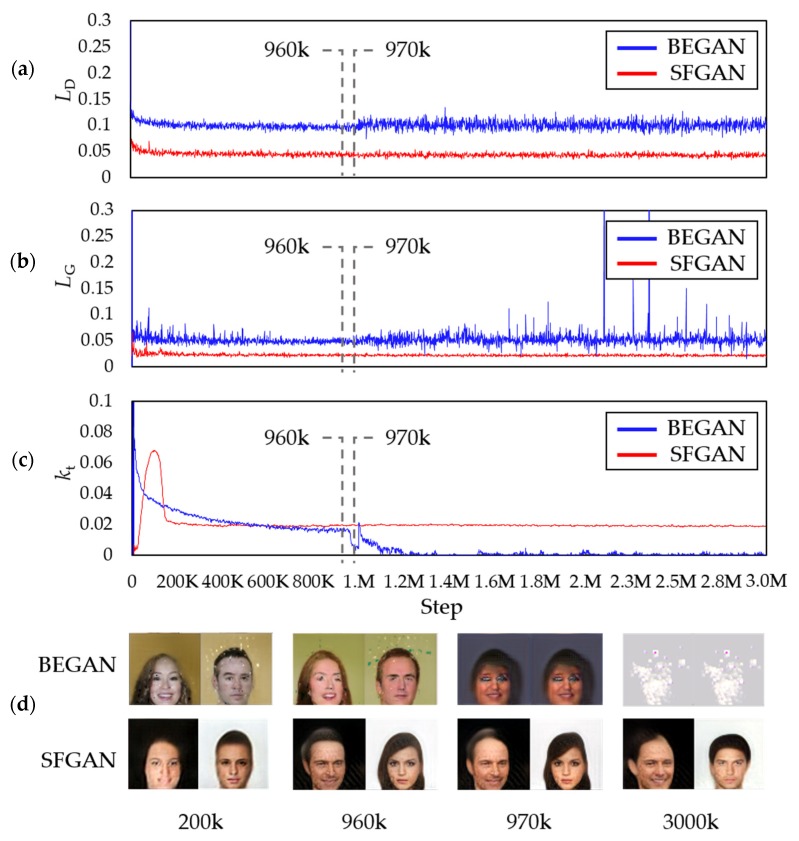
Comparison of BEGAN and SFGAN trained with CelebA dataset in 64 × 64 resolution: (**a**) Discriminator loss $L_{D}$; (**b**) generator loss $L_{G}$; (**c**) variable $k_{t};$ (**d**) samples during the training process of BEGAN and SFGAN with α=12,288.

Figure 8 compares the two losses, $k_{t}$s, and the generated images during the training process of the two models. Figure 8a,b shows the losses of discriminator and generator of the models, respectively. The amplitudes of both losses of discriminator and generator of BEGAN increase after 970k steps, while those of SFGAN do not change much and even seem to converge. The loss differences for the two models appeared the difference between generated images, as shown in Figure 8d. That is, the generator of BEGAN fails to generate human-like faces at 970k step, while the generator of SFGAN keeps on generating human-like faces until 3000k steps. Figure 8c shows that the change of $k_{t}$ is negligible if GANs are in the equilibrium state. That is, the more stable the training gives a smaller variation of $k_{t}$ [50]. In early training stages, both of BEGAN and SFGAN are tending to generate easy-to-reconstruct data by auto-encoder because the real data distribution has not been learned accurately yet. BEGAN seems to find a stable value as it gradually descends. When BEGAN started to generate images that are not human-like faces, however, $k_{t}$ decreases rapidly. This means that the discriminator’s loss is reduced faster than that of the generator. In other words, the discriminator won the generator. SFGAN, on the other hand, $k_{t}$ increases and then decreases slowly until 150,000 steps. After 150,000 steps, no abrupt decrements are observed for SFGAN as BEGAN does have. Based on this observation, we may derive the following: (1) If $k_{t}$ is not zero and has small vibration or converging to a constant that is not zero, then the network can be considered as in a stable state. (2) If $k_{t}$ converges to zero, the generator’s loss is too large, and the network is far from the equilibrium state.

#### 5.2.2. The Generated Images of CelebA: 128 × 128 Resolution

**Figure 9:**
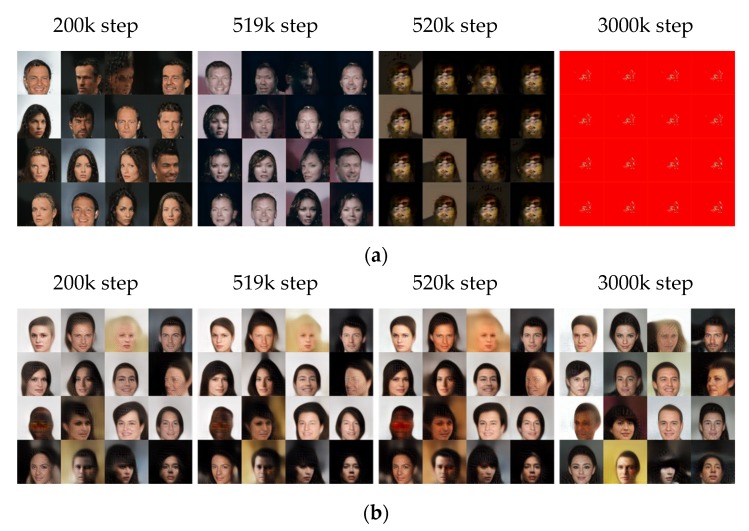
A total of 16 samples generated at 200k, 519k, 520k, and 3000k steps trained with CelebA dataset in 128 × 128 resolution by: (**a**) BEGAN; (**b**) SFGAN with α=12,288.

Figure 9 presents the results for the images with 128 × 128 resolution. In the 200k step, the training result of BEGAN is similar to that of SFGAN. However, it is observed that the mode collapse occurs 520k, which is earlier than that with 64 × 64 resolution in BEGAN. At 3000k step, BEGAN generated the same images even if different inputs are given. Based on the results with two different resolutions, the mode collapse seems to occur faster as the image resolution increases in BEGAN. This phenomenon seems caused by the insufficiency of the weight parameters in the model network because the mode collapse and/or mode drop can easily occur when the number of weight parameters is insufficient [63].

**Figure 10:**
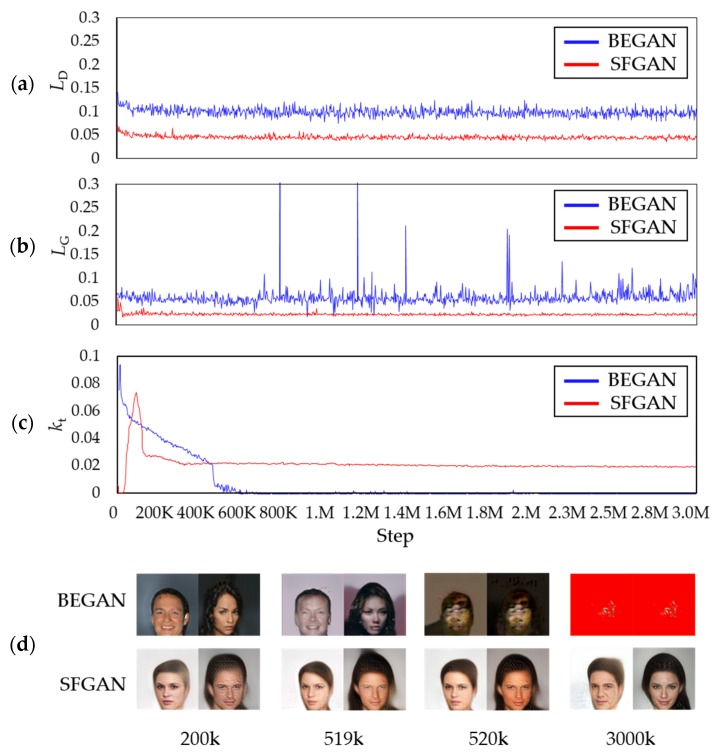
Comparison of BEGAN and SFGAN trained with CelebA dataset in 128 × 128 resolution: (**a**) Discriminator loss $L_{D}$; (**b**) generator loss $L_{G}$; (**c**) variable $k_{t};$ (**d**) samples during the training process of BEGAN and SFGAN with α=12,288.

Figure 10 compares the two losses, $k_{t}$s, and the generated images during the training process of the two models. Figure 10a,b show that the differences in both discriminators’ losses are insignificant, while those of both generators’ losses seem significant. The loss of generator of BEGAN fluctuates significantly after 700k steps compared to the previous steps, while the loss of generator of SFGAN is almost consistent. The instant of the abrupt changes in loss of generator with this resolution is different from that with a smaller resolution, 520k steps. This can be interpreted in two ways; (i) The exact instant is missing because the losses are measured at every 1000 steps. (ii) The mode collapse and/or mode drop can occur even if the generator learns stably. Figure 10c illustrates $k_{t}$s for every 1000 steps of the two models, and Figure 10d presents the created samples corresponding to 200k steps, 519k steps, 520k steps, and 3000k steps. At 520k steps, $k_{t}$ of BEGAN dropped rapidly, and the similar images are generated. The rapid drop of $k_{t}$ implies that the loss of discriminator does not change much during its update, and this may result in the model collapse or mode drop. This phenomenon did not occur in SFGAN until 3000k steps, as same as 64 × 64 resolution.

If we compare the results of two resolutions and two models, the followings are concluded; (i) BEGAN requires one and two more convolutional layers for the generator and discriminator, respectively, if the resolution becomes twice. (ii) The used network architecture is not a good model for large sizes of images. (iii) Even though the network structure of SFGAN is the same as BEGAN, it is less affected by the architecture and, therefore, resolution. This phenomenon seems to owe to using SFD.

#### 5.2.3. The Generated Images of CIFAR-10: 32 × 32 Resolution

**Figure 11:**
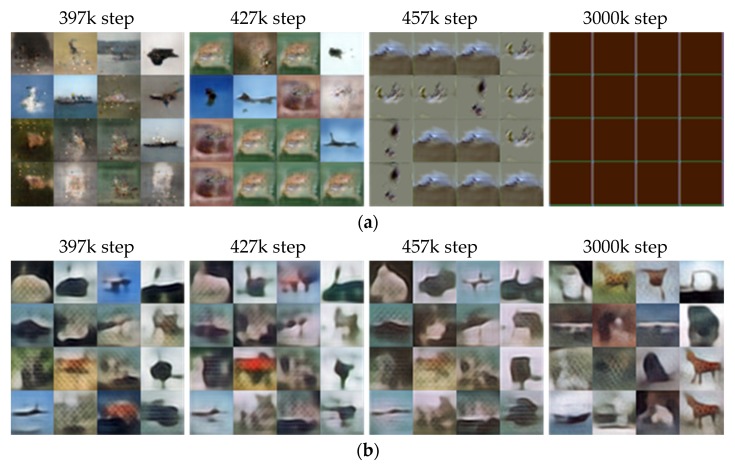
A total of 16 samples generated at 397k, 427k, 457k, and 3000k steps trained with CIFAR-10 dataset by: (**a**) BEGAN; (**b**) SFGAN with α=12,288.

**Figure 12:**
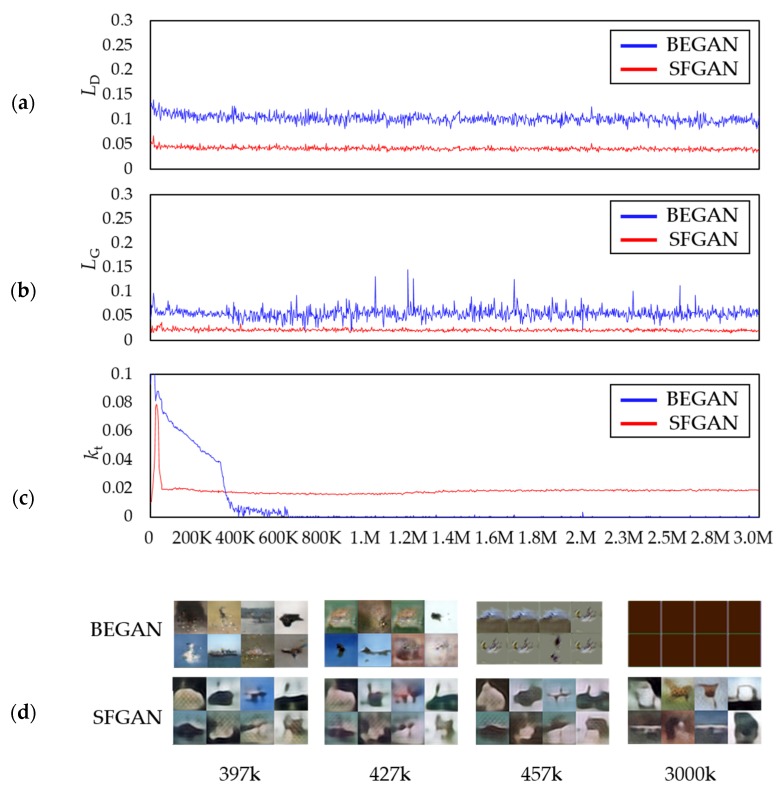
Comparison of BEGAN and SFGAN trained with CIFAR-10 dataset: (**a**) Discriminator loss $L_{D}$; (**b**) generator loss $L_{G}$; (**c**) variable $k_{t};$ (**d**) samples during the training process of BEGAN and SFGAN with α=12,288.

The same experiments are conducted with CIFAR-10. Figure 11 and Figure 12 present the results with 32 × 32 resolution. Figure 11 compares the images generated by the two models at 397k, 427k, 457k, and 3000k steps. Overall, it is difficult to figure out the images created by both models. According to [63], BEGAN-based models perform slightly better than DCGAN in training CIFAR-10. Therefore, it can be derived that the obtained unclear images may be caused by the network structure of BEGAN. The figure shows that BEGAN seems to produce relatively sharp images initially up to 397k steps compared to SFGAN. However, similar images are generated from 427k, and it seems to fail to generate different images from 457 k steps. The generated images are all the same images at 3000k steps finally. On the other hand, SFGAN generates different images continuously, even though the images are blurry from the beginning. Figure 12 compares the two losses, $k_{t}$s, and the generated images during the training process of the two models. The losses of discriminator seem stable in both models, while the losses of the generator are not. That is, the loss of generator in BEGAN increases slightly from about 400k steps and then fluctuates after all. This phenomenon may result in a lack of diversity in the generated images.

On the other hand, for SFGAN, the amplitude of loss of the generator gradually decreases, staying around small values near zero. As shown in Figure 12c, the $k_{t}$ values for BEGAN drop rapidly at 397k steps, 427k steps, and 457 steps, which seems to be associated with the reduction of diversity in the generated images, as shown in Figure 12d. The $k_{t}$s for SFGAN does not drop abruptly, except in the initial stage of training. The stable $k_{t}$s seem to correspond to the generated images of SFGAN.

Comparing the results of two datasets, CelebA and CIFAR-10, the followings are concluded: (i) BEGAN and SFGAN can make human faces up to 500k steps when training a CelebA dataset, but it is not valid for the CIFAR-10. This phenomenon seems to owe to the number of training data because the number of images of CelebA is approximately 3.4 times that of CIFAR-10. (ii) Based on the training results of CIFAR-10, the capacities of SFD and BED as distance metrics are similar in training, while SFD is better than EMD in stability and performance.

### 5.3. Mixture of Gaussian Dataset

**Figure 13:**
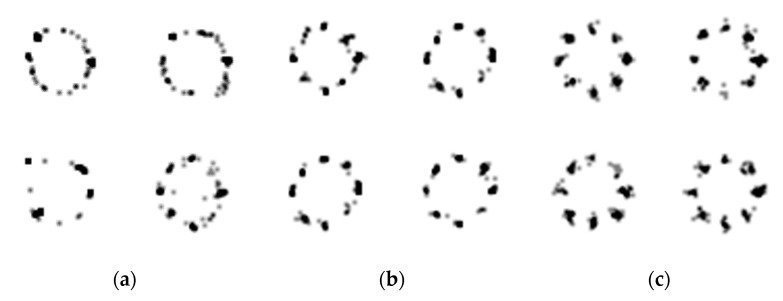
Four batches of 100 sample generation results trained with a 2-D mixture of Gaussian by: (**a**) BEGAN; (**b**) SFGAN with α=12,288; (**c**) training data.

In this section, we compare the qualitative results of a mixture of Gaussian. Figure 13c shows examples of images used as training data. The example images are dawn by randomly generated 100 samples from the mixture of Gaussian distribution. Figure 13a,b presents the generated images by BEGAN and SFGAN, respectively. As shown in the figure, both models learn roughly the circular positions of the random variables. However, the densities of each eight centers of BEGAN are relatively low compared to those of SFGAN. These results imply that SFD enables us to learn the distribution at least as same as or better than EMD.

### 5.4. Detecting Mode Collapse Using Distances

**Figure 14:**
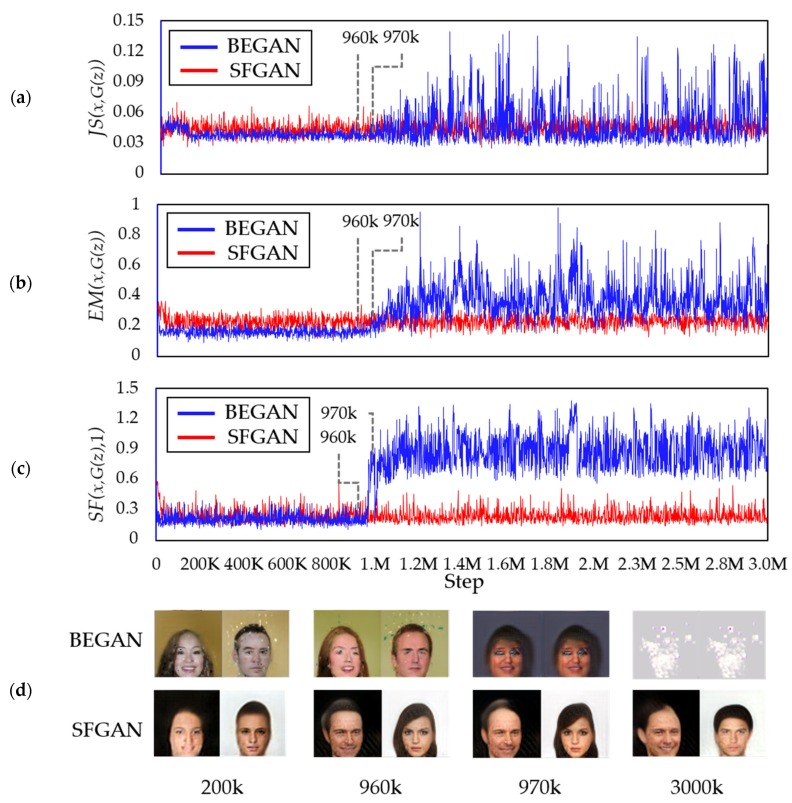
Comparison of distances between the distributions of training data and data generated by BEGAN and SFGAN: (**a**) JSD; (**b**) EMD; (**c**) SFD with α=1; (**d**) generated samples of the two models.

Figure 14 compares (a) JSD, (b) EMD, (c) SFD between the training data and the generated data for every 1000 steps during the training process of BEGAN and SFGAN with α=12,288 for (a) and (b), and α=1 for (c), and (d) the generated samples presented in Figure 14 d. At a glance, each of the three distance metrics for BEGAN is more fluctuate than that of SFGAN, especially after 970k. For BEGAN, the following are observed; For JSD, it is difficult to find the exact spot where the distance is distinguished from detecting the mode collapse even though the amplitude of the values increases between 960k and 970k. For EMD, the range of distances slightly increases from 960k to 970k after mode collapse occurs. However, there are overlapping ranges before and after the mode collapse occurs, which seems to owe the definition of EMD. In the case of SFD, however, the ranges of distance differ significantly before and after the mode collapse, compared to those of JSD and EMD. In other words, mode collapse and/or mode drop detection is detected better by SFD than by JSD and EMD. As already seen in Section 5.2, no collapse occurs in SFGAN, and distance metrics verifies this. As a result, the distance values of SFGAN in (a), (b), and (c) can imply the stable state.

In Table 4, the mean values of the three distance metrics are compared for two groups: a group from 1 step to 968k step and a group from 969k step to three million steps, for the two models. The presented values in the table are the averages of ten experiments. In BEGAN, all values of JSD, EMD, and SFD for the second group are increased compared to the corresponding value for the first group. The biggest increment is observed in SFD, followed by JSD and EMD. This increment shows that mode collapse and/or mode drop signs can be captured without the inception v3 model or balanced dataset, which are regarded requirements for detecting these phenomena. All values of the three metrics for SFGAN are slightly increased in the second group compared to the corresponding value for the first group, even if mode drop and (or) mode collapse did not yet appear. This phenomenon can be interpreted as two situations: (1) SFGAN’s learning is almost balanced, which derives similar values of distances. (2) SFGAN remains the possibility to occur mode drop and (or) mode collapse.

### 5.5. Quantitative Comparison

The performance of SFGAN is evaluated in two ways; comparison of FID index with BEGAN and comparison with well-known GAN models.

**Figure 15:**
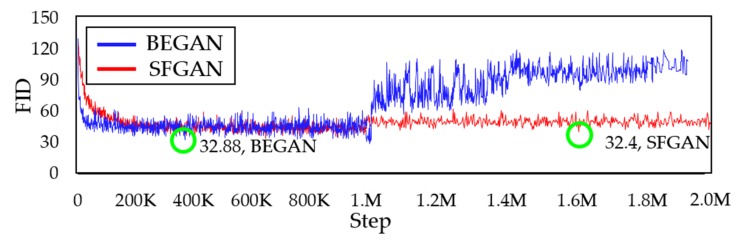
Comparison of FID between the training data and the generated data by BEGAN and SFGAN.

Figure 15 shows the FID [20] between the real and the generated data for BEGAN and SFGAN for every 1000 steps up to 2000k steps. As the green circle indicates, the minimum values of FID for both models are 32.88 and 32.4, respectively, which are almost the same. The FID values of BEGAN increased suddenly around 970k, while those of SFGAN remain steady. However, BEGAN could no longer maintain its quality after 970k, while SFGAN maintained its quality until 2000k steps. This FID value demonstrated that SFGAN is not in mode drop or mode drop phenomenon.

The obtained FID indices of the two models are compared with those of well-known GAN models. Table 5 summarizes the FID indices of those GAN models, including SFGAN. The FID indices of DCGAN, WGAN, and WGAN-GP in the table are from [28], which obtained by using CelebA and CIFAR-10.

For the CelebA dataset, WGAN-GP performs best. This superiority comes from the process of calculating the gradient of the discriminator. The calculation of gradient in WGAN-GP is executed the forward and backward propagation as a whole. Even though SFGAN is in the second position, it is the best FID value in the models that do not calculate the gradient. The models with BEGAN-based architecture, such as BEGAN and SFGAN, appear to be better models for learning CelebA data than the models with DCGAN-based architecture. Note that DCGAN, WGAN, and WGAN-GP belong to models with DCGAN-based architecture.

Meanwhile, when training CIFAR-10, BEGAN and SFGAN are worse than WGAN and WGAN-GP. These performances are because the models with BEGAN-based architecture do not train the dataset sufficiently, which seems to owe the number of filters of a layer [63]. In other words, it is assumed that the number of filters in each layer within the BEGAN-based architecture is smaller than that of the DCGAN-based architecture.

Even though the training processes of some experiments turn out to be unstable and the same or blurred images are generated, it is difficult to figure out the reason for the results. Maybe it is because of mode drop or mode collapse or both. At the current level of researches, it is not very easy to find out the relationship between the stability of the training process and the two phenomena, quantitatively measure and distinguish the two phenomena.

## 6. Conclusions

We have introduced a distance metric SFD and proposed a SFGAN model. SFD has used for two cases: one is in loss functions of SFGAN, and the other is as a measure to detect mode drop and mode collapse during the training process. SFGAN has implemented using several datasets, including CelebA and CIFAR-10, and is compared with BEGAN that is using auto-encoder and EMD. Experimental results show that the training process of SFGAN is more stable than that of BEGAN under the same conditions. Also, it is verified that SFD is an acceptable distance metric presenting better results than the existing distance metrics such as JSD and EMD in detecting mode drop and/or mode collapse. This study will be extended to apply SFD in the field of GAN and apply SFGAN to various datasets such as ImageNet. The relationship between stability and mode collapse and/or mode drop and how to distinguish between mode collapse and mode drop will be studied in the future.

## Figures and Tables

**Table 1:** Comparison of JSD, EMD, and SFD for the examples.

Distance Metric	Example 1	Example 2
Jensen–Shannon distance (JSD)	$\begin{array}{cc} \log2, & \theta \neq 0\\ 0, & \theta =0 \end{array}$	$\frac{1}{2}\log\frac{\delta^{2}+1}{2\delta }$
Earth Mover’s distance (EMD)	|θ|	[0,|δ−1|]
Simplified Fréchet distance (SFD)	|θ|	$\frac{1}{\sqrt{\alpha }}\left|\delta -1\right|$

**Table 2:** Procedure of SFGAN.

The Procedure of SFGAN
1. Generate n noise samples $z=\{z^{1},\cdots ,z^{n}\}.$ Each $z^{i}$ is vector given by $z^{i}=(z_{1}^{i},z_{2}^{i},\ldots ,z_{d}^{i})$, where *d* is the number of dimensions, and each component of $z^{i}$ is randomly generated according to a uniform distribution in the interval [−1,1] with pdf $p_{z}.$
2. The generator G generates fake data G(z) based on the generated samples z.
3. n samples $x=\{x^{1},\cdots ,x^{n}\}$ are taken from real data with pdf $p_{data}.$
4. Auto-encoder D receives G(z) and x as inputs and produces D(G(z)) and D(x) as outputs.

**Table 3:** Hyperparameters used in the experiments.

Hyperparameter	Value
Batch size (n)	64
CelebA resolutions (h×w)	64 × 64, 128 × 128
CIFAR-10 resolution (h×w)	32 × 32
Channel unit (c)	64
Regularization coefficient for training (α)	12,288
Regularization coefficient for detecting (α)	1
Adam ($\beta_{1},\beta_{2}$)	0.9, 0.999
Proportional gain ($\lambda_{k}$)	0.001
Diversity ratio (γ)	0.5
Learning rate	0.001
Total global steps	3000,000

**Table 4:** Mean values of JSD, EMD, and SFD of two groups; Before 968k (from 1 step to 968k) and After 968k (from 969k step to three million steps) for BEGAN and SFGAN.

Model	BEGAN		SFGAN	
	Step Group		Step Group	
	Before 968k	After 968k	Before 968k	After 968k
JSD	0.038065	0.048083	0.044120	0.045827
EMD	0.152573	0.365471	0.227430	0.229367
SFD	0.197894	0.867904	0.237440	0.240151

**Table 5:** Comparison of FID indices by several GAN models.

Model	CelebA	CIFAR-10
DCGAN	65.6 ± 4.2	72.7 ± 3.6
WGAN	41.3 ± 2.0	55.2 ± 2.3
WGAN-GP	30.0 ± 1.0	55.8 ± 0.9
BEGAN	38.9 ± 0.9	71.4 ± 1.6
SFGAN	38.1 ± 0.8	68.4 ± 1.3

## Appendix A

**Definition** **A1.**
*The Jensen–Shannon distance (JSD).*

(A1) $$JS(F,G)=\frac{1}{2}KL\left(F\|\frac{F+G}{2}\right)+\frac{1}{2}KL\left(G\|\frac{F+G}{2}\right),$$

where KL(F‖G) is the Kullback–Leibler (KL) divergence defined by

(A2) $$KL(F\|G)=\int f(x)\log\left(\frac{f(x)}{g(x)}\right)dx,$$

where both f(x) and g(x) are absolutely continuous with $F(A)=\int_{A}f(x)dx$ and $G(A)=\int_{A}g(x)dx$ [18].

JSD is based on the KL divergence considering symmetric property which KL divergence does not have. Since JSD is bounded and symmetric [64], it is the first distance metric applied in GANs. However, it has the disadvantage of not being able to learn the data distribution in low dimension [15].

**Definition** **A2.** *The Earth-Mover distance (EMD or Wasserstein-1)*.

(A3) $$EM(F,G)=\underset{\gamma \in \Theta }{\inf}E_{(X,Y)\sim \gamma }[|X-Y|],$$

where Θ is the set of all joint distributions whose marginal distributions of X and Y are F and G, respectively. The EMD considers the minimal cost for transformation of distributions.

**Definition** **A3.**
*Maximum Mean Discrepancy (MMD)*

(A4) $$MMD(\psi ,F,G)=\underset{f\in \psi }{\sup}\left(E_{x\sim F}\left[f\left(x\right)\right]-E_{y\sim G}\left[f\left(y\right)\right]\right),$$

where ψ={h:χ→ℝ:χ is a metric space} and *F* and *G* are the distribution functions of x and y,x,y∈χ, respectively [44].

## Appendix B

For Example 1, the JSD, EMD, and SED are computed as follows:

**Lemma** **A1.**

(A5) $$(a)JS(F,G)=\{\begin{array}{cc} \log2, & \theta \neq 0,\\ 0, & \theta =0. \end{array}$$

(A6) (b)EM(F,G)=|θ|

(A7) (c)SF(F,G,α)=|θ|

**Proof.** (a) If θ=0, then F and G are the same distribution. Therefore, $\frac{F+G}{2}=F=G$ and this gives

(A8) $$JS(F,G)=\frac{1}{2}KL\left(F\|F\right)+\frac{1}{2}KL\left(F\|F\right)=KL\left(F\|F\right)=\int f(x)\log\left(\frac{f(x)}{f(x)}\right)dx=\int f(x)\cdot 0dx=0$$

If θ≠0, then

(A9) $$\begin{array}{ll} JS(F,G) & =\frac{1}{2}KL\left(F\|\frac{F+G}{2}\right)+\frac{1}{2}KL\left(G\|\frac{F+G}{2}\right)\\ & =\frac{1}{2}\int f(x)\log\left(\frac{2f(x)}{f(x)+g(x)}\right)dx+\frac{1}{2}\int g(x)\log\left(\frac{2g(x)}{f(x)+g(x)}\right)dx\\ & =\frac{1}{2}\int f(x)\log\left(\frac{2f(x)}{f(x)}\right)dx+\frac{1}{2}\int g(x)\log\left(\frac{2g(x)}{g(x)}\right)dx\\ & =\frac{\log2}{2}\int f(x)dx+\frac{\log2}{2}\int g(x)dx\\ & =\log2. \end{array}$$

(b) By Jensen’s inequality, the following relation holds.

(A10) $$\underset{\gamma \in \Theta }{\inf}\left|E_{(X,Y)\sim \gamma }\left[X-Y\right]\right|\leq \underset{\gamma \in \Theta }{\inf}E_{(X,Y)\sim \gamma }\left[\left|X-Y\right|\right]\leq \underset{\gamma \in \Theta }{\inf}\sqrt{E_{(X,Y)\sim \gamma }{\left\|X-Y\right\|}^{2}}.$$

That is,

(A11) $$\underset{\gamma \in \Theta }{\inf}\left|E_{(X,Y)\sim \gamma }[X]-E_{(X,Y)\sim \gamma }[Y]\right|\leq EM\left(F,G\right)\leq \sqrt{Fr^{2}\left(F,G\right)}$$

and this gives

(A12) $$\underset{\gamma \in \Theta }{\inf}\left|m_{X}-m_{Y}\right|=\left|\theta \right|\leq EM\left(F,G\right)\leq \sqrt{Fr^{2}\left(F,G\right)}.$$

FD, the upper bound of Equation (A10), for this example is as follows:

(A13) $$Fr^{2}\left(F,G\right)=\theta^{2}+\mathrm{tr}\left(\left(\begin{array}{cc} 0 & 0\\ 0 & 1 \end{array}\right)+\left(\begin{array}{cc} 0 & 0\\ 0 & 1 \end{array}\right)-2{\left(\left(\begin{array}{cc} 0 & 0\\ 0 & 1 \end{array}\right)\left(\begin{array}{cc} 0 & 0\\ 0 & 1 \end{array}\right)\right)}^{\frac{1}{2}}\right)=\theta^{2}.$$

Therefore, EM(F,G) is given by

(A14) EM(F,G)=|θ|.

(A15) $$(c)SF^{2}\left(F,G,\alpha \right)=\theta^{2}+\frac{1}{\alpha }\cdot 0^{2}=\theta^{2}.$$

 □

The JSD, EMD, and SFD for Example 2 are computed as follows.

**Lemma** **A2.**

(A16) $$(a)JS(F,G)=\frac{1}{2}(\log\frac{\delta^{2}+1}{2\delta }),$$

(A17) (b) 0≤EM(F,G)≤|δ−1|,

(A18) $$(c)SF\left(F,G,\alpha \right)=\frac{1}{\sqrt{\alpha }}\left|\delta -1\right|.$$

**Proof.** (a) Since F and G are belonging to the same mixture family, JS(F,G) can be expressed as a Jensen–Bregman divergence [65]. Therefore, it can be written as:

(A19) $$JS(F,G)=\frac{1}{2}\left(\frac{1}{2}m_{X}^{Τ}C_{X}^{-1}m_{X}+\frac{1}{2}m_{Y}^{T}C_{Y}^{-1}m_{Y}-m_{1/2}^{T}C_{1/2}^{-1}m_{1/2}+\log\frac{{\left|C_{X}\right|}^{1/2}{\left|C_{Y}\right|}^{1/2}}{\left|C_{1/2}\right|}\right),$$

where $C_{1/2}={\left(\frac{1}{2}C_{X}^{-1}+\frac{1}{2}C_{Y}^{-1}\right)}^{-1}$ and $m_{1/2}=C_{1/2}\left(\frac{1}{2}C_{X}^{-1}m_{X}+\frac{1}{2}C_{Y}^{-1}m_{Y}\right).$ Substituting the mean and covariance of X and Y respectively, $C_{1/2}$ and $m_{1/2}$ are simply written as

(A20) $$C_{1/2}={\left(\frac{1}{2}1+\frac{1}{2}\delta^{-2}\right)}^{-1}=\frac{2\delta^{2}}{\delta^{2}+1}\;\mathrm{and}\;m_{1/2}=C_{1/2}\left(\frac{1}{2}\cdot 1\cdot 0+\frac{1}{2}\cdot \delta^{-2}\cdot 0\right)=\frac{2\delta^{2}}{\delta^{2}+1}\cdot 0=0,$$

respectively. Therefore, the JSD between *F* and *G* is computed as

(A21) $$JS(F,G)=\frac{1}{2}\left(\frac{1}{2}0\cdot 1\cdot 0+\frac{1}{2}0\cdot \delta^{-2}\cdot 0-0\cdot \frac{\delta^{2}+1}{2\delta^{2}}\cdot 0+\log\frac{1^{1/2}{\left|\delta^{2}\right|}^{1/2}}{\left|\frac{2\delta^{2}}{\delta^{2}+1}\right|}\right)==\frac{1}{2}\left(\log\frac{\delta^{2}+1}{2\delta }\right)$$

(b) By Equation (A10), we obtain

(A22) $$\underset{\gamma \in \Theta }{\inf}\left|m_{X}-m_{Y}\right|=0\leq EM\left(F,G\right)\leq \sqrt{Fr^{2}\left(F,G\right)}.$$

In this example, $m_{X}=m_{Y}=0$, $C_{X}=1^{2}$, and $C_{Y}=\delta^{2}$. Therefore, FD, the upper bound of Equation (A22), is as follows:

(A23) $$Fr^{2}\left(F,G\right)=1^{2}+\delta^{2}-2{\left(1^{2}\delta^{2}\right)}^{\frac{1}{2}}={\left(\delta -1\right)}^{2}.$$

Finally, EM(F,G) is given by

(A24) $$\underset{\gamma \in \Theta }{\inf}\left\|0-0\right\|=0\leq EM\left(F,G\right)\leq \left|\delta -1\right|.$$

(c) In this example, $m_{X}=m_{Y}=0$, $\sigma_{X}=1^{2}$, and $\sigma_{Y}=\delta^{2}$, therefore, $SF^{2}\left(F,G,\alpha \right)$ is computed as

(A25) $$SF^{2}\left(F,G,\alpha \right)={\left\|m_{X}-m_{Y}\right\|}^{2}+\frac{1}{\alpha }{\left\|\sigma_{X}^{}-\sigma_{Y}^{}\right\|}^{2}={\left\|0-0\right\|}^{2}+\frac{1}{\alpha }{\left\|1-\delta \right\|}^{2}$$

and this gives the result. □
